# Consistency of noncognitive skills and their relation to educational outcomes in a UK cohort

**DOI:** 10.1038/s41398-021-01661-8

**Published:** 2021-11-05

**Authors:** Tim T. Morris, George Davey Smith, Gerard van den Berg, Neil M. Davies

**Affiliations:** 1 MRC Integrative Epidemiology Unit, University of Bristol, Bristol, UK; 2 Population Health Sciences, Bristol Medical School, University of Bristol, Barley House, Oakfield Grove, Bristol, UK; 3 Department of Economics, University of Bristol, Priory Road Complex, Bristol, UK; 4 Department of Economics, University of Groningen, Groningen, The Netherlands; 5 Department of Epidemiology, University Medical Center Groningen, Groningen, The Netherlands

**Keywords:** Human behaviour, Genomics

## Abstract

Noncognitive skills have been shown to associate with a range of health and socioeconomic outcomes. Many studies have relied on cross sectional data and have been unable to assess the longitudinal consistency of noncognitive skill measures. Using data from a UK birth cohort, we investigated a range of noncognitive skills: behavioural problems, social skills, communication, self-esteem, persistence, locus of control, empathy, impulsivity and personality. We assessed their consistency over a 17-year period throughout childhood and adolescence (age 6 months to 18 years), their genomic architecture, and their associations with socioeconomic outcomes. We found high longitudinal measurement consistency for behavioural and communication skills, but low consistency for other noncognitive skills, suggesting a high noise to signal ratio. We observed consistent non-zero heritability estimates and genetic correlations for only behavioural difficulties. Using aggregate measures of each skill over time, we found evidence of phenotypic correlations and heritability ($h_{SNP}^2$ = 0.1–0.2) for behaviour, communication, self-esteem and locus of control. Associations between noncognitive skills and educational outcomes were observed for skills measured in mid to late childhood but these were at most a third of the size of IQ-education associations. These results suggest that measures designed to capture noncognitive skills may be subject to considerable response heterogeneity or measurement error. Aggregate measures that leverage repeat responses from longitudinal data may offer researchers more reliable measures that better identify underlying noncognitive skills than cross sectional measures.

## Introduction

Noncognitive (or socioemotional or “soft”) skills are broadly defined as “personality traits, goals, character, motivations, and preferences” [1] that represent patterns of behaviour [2]. They are considered complementary and related to cognitive measures such as intelligence or general cognitive ability [2, 3]. A wide range of characteristics have been proposed as noncognitive skills, including multifaceted personality and behavioural traits such as persistence, motivation, temperament, attention, communication, confidence and self-esteem (see Supplementary Box 1 for a list of characteristics previously considered as noncognitive) [3–7]. Correlations between these traits [8–10] supports the notion of a broad multidimensional definition of noncognitive skills. Here, we consider noncognitive skills using the definitions provided above as *personality traits, goals, character, motivations, and preferences that represent patterns of thought, feelings, and behaviour*.

Noncognitive skills have been posited as an important driver of individual differences for a range of socioeconomic outcomes including educational attainment and job performance, though the evidence base is diverse and inconsistent [1, 3, 4, 7, 9, 11–18]. Associations between the Big Five personality types (extraversion, agreeableness, conscientiousness, neuroticism and openness) and outcomes replicate well [19], but a recent systematic review showed that many other character traits defined as noncognitive skills have small and heterogenous effects across studies [20]. Few studies have measured a wide battery of noncognitive skills [17, 18], making comparisons between these skills difficult. Few have also adjusted estimates for cognitive ability, despite its strong attenuating effect on non-cognitive associations with socioeconomic outcomes [15, 16]. It is therefore not well known how well non-cognitive skills in childhood associate with each other.

Given that many studies have been conducted on samples with similar age ranges [20], these heterogenous effects may arise from within-individual variation over time, measurement error, or noncognitive skills not being robust constructs. However, short follow-up and a lack of longitudinal data has made it difficult to estimate the consistency with which noncognitive skills are measured [20, 21]. Test-retest reliability (an indicator of measurement accuracy) has been estimated at 0.31 for personality in childhood, 0.12 to 0.61 for risk aversion, and 0.49 for locus of control [22–24]. These values are lower than those reported for cognitive skills (c.f. 0.52 for digit span to 0.82 for reading) [23], suggesting that the instruments used to measure noncognitive skills are likely to be characterised by greater variation over time or greater measurement error than the instruments used to measure cognitive skills [1]. While it is difficult to disentangle within-individual variation from measurement consistency, true within-individual variation over time can be expected to be characterised by decreased intra-person correlations with larger periods of elapsed time, while measurement error can be expected to be characterised by consistent intra-person correlations.

The measurement consistency of noncognitive skills over time and the robustness of their associations with socioeconomic outcomes can be explored with genetic data. Identifying signal in genetic analyses requires the phenotype to have a genotypic component. Personality types have been the most studied of the noncognitive skills with heritability estimated at ~ 0.5 [25]. Heritability estimates for other noncognitive skills has varied from 0.44 to 0.79 for self-control [26, 27]; 0.36 for alienation [9]; 0.83 for academic effort [9]; 0.31 to 0.56 for aspects of openness [28]; 0.18 to 0.49 for aspects of conscientiousness [29]; and 0.40 for enjoyment and self-perceived ability [30]. There is also genetic evidence that personality types are stable over time [31], providing support that personality measures are sufficiently free from measurement error to isolate a genetic signal. However, many studies have been unable to investigate the genetics of multiple noncognitive skills within the same sample of individuals [32].

In this study, we contribute to the literature with a comprehensive analysis of several noncognitive skills using data from a UK cohort study. We investigate the relationships between 9 measures of noncognitive skills, educational achievement, and labour market outcomes to answer two related research questions: (1) How consistent are noncognitive skills measured in a large cohort study over 18 years? (2) How strongly do different noncognitive skills associate with socioeconomic outcomes?

## Materials and methods

### Study sample

Participants were children from the Avon Longitudinal Study of Parents and Children (ALSPAC). Pregnant women resident in Avon, UK with expected dates of delivery 1st April 1991 to 31^st^ December 1992 were invited to take part in the study. Two phases of recruitment resulted in a total sample of 14,899 children who were alive at one year of age, of whom 7988 had genetic data. For full details of the cohort profile and study design see [33, 34]. The ALSPAC cohort is largely representative of the UK population when compared with 1991 Census data; there is under representation of some ethnic minorities, single parent families, and those living in rented accommodation [33]. Ethical approval for the study was obtained from the ALSPAC Ethics and Law Committee and the Local Research Ethics Committees. All methods were performed in accordance with the relevant guidelines and regulations. Informed consent for the use of data collected via questionnaires and clinics was obtained from participants following the recommendations of the ALSPAC Ethics and Law Committee at the time. Consent for biological samples has been collected in accordance with the Human Tissue Act (2004). We used the largest available samples in each of our analyses to increase precision of estimates, regardless of whether a child has data on other noncognitive skills (see Supplementary Table 1 for sample sizes).

### Genetic data

DNA of the ALSPAC participants was extracted from blood, cell line and mouthwash samples, then genotyped using references panels and subjected to standard quality control approaches. Briefly, genotype data for the ALSPAC children was measured using the Illumina HumanHap550 quad chip genotyping platforms and then combined with genotype data for the ALSPAC mothers to improve imputation accuracy, which was measured using Illumina human660W-quad array platforms. Only children’s genotype data was used in analyses. All individuals with non-European ancestry were removed and exclusions were made based on gender mismatches; minimal or excessive heterozygosity; disproportionate levels of individual missingness (>3%); insufficient sample replication (IBD < 0.8); low SNP frequency (<1%), call rate (<95%) and violations of Hardy-Weinberg equilibrium (*P* < 5E-7). Genotypes in common were combined and imputed to the Haplotype Reference Consortium (HRCr1.1, 2016) panel of approximately 31 000 phased whole genomes, giving 8,237 eligible children with available genotype data after exclusion of related individuals using cryptic relatedness measures. Principal components were generated by extracting unrelated individuals (IBS < 0.05) and independent SNPs with long range LD regions removed. For full details of genotyping see the Supplementary Material.

### Noncognitive skills

We used all personality traits that have previously been considered in the literature as noncognitive skills (Supplementary Box 1) that were available in the ALSPAC data, had at least 1,000 responses, and where binary had at least 100 responses in each category. The supplementary material contains more detailed information on the measures used, their components and a timeline of when all skills and outcomes were measured (Supplementary Fig. 1).

### SDQ

The Strengths and Difficulties Questionnaire (SDQ) is used to assess child emotional and behavioural difficulties. It consists of five items measured across each of five scales that cover common areas of emotional and behavioural difficulties; emotional symptoms, conduct problems, hyperactivity/inattention, peer relationship problems, and prosocial behaviour. Study mothers reported on the SDQ for children on seven occasions at child ages 4, 7, 8, 10, 12, 13 and 16 years, and the children’s teachers completed an SDQ questionnaire for each child at ages 7 and 10. We use the total difficulties score, which is defined as the count of problems on the first four scales [35]. To ensure that our results are not biased by potential inverse associations of internalising and externalising factors with socioeconomic outcomes, we ran sensitivity analyses using the internalising (emotional symptoms and peer relationship problems) and externalising (conduct problems and hyperactivity/inattention) sub-scales separately [36]. We use the SDQ as a measure of emotional and behavioural regulation [20, 37]. All SDQ scores were reverse coded so that high values refer to fewer problems.

### Social skills

Social skills at age 13 were determined using a battery of 10 questions reported by the study mother. Responses were reported on a five-point scale and then summed to provide a total overall social skills score.

### Communication

Communication at 6 months was calculated from mother-reported responses to a battery of eight questions asking about the development of their child’s communication skills. At age 1 communication was calculated from mother-reported responses using response to 82 questions on the MacArthur Infant Communication questionnaire. At 18 months communication was calculated using mother-reported responses to a battery of 14 questions asking about the development of their child’s communication skills. At age 3 communication was calculated from mother-reported responses to a battery of 123 questions forming a vocabulary score. At age 10 communication was calculated from mother-reported responses as the sum of five domains of communication from a total battery of 39 questions.

### Self-esteem

Self-esteem at age eight was measured using self-report responses to the 12-item shortened form of Harter’s Self Perception Profile for Children, comprising the global self-worth and scholastic competence subscales. Self-esteem at age 18 was measured using self-report responses to 10 questions of the Bachman revision of the Rosenberg Self-Esteem Scale.

### Persistence

Persistence at age 6 months was measured as a weighted score from mother-reported responses to seven questions relating to child temperament. At age 2, persistence was measured as a weighted score from nine mother-reported responses. At age 7, persistence was recorded by ALSPAC interview testers as the study child’s persistence when completing a direct assessment session in which participants had to match words and pictures, with responses categorised as persistent and sometimes persistent or not persistent.

### Locus of control

Locus of control, the strength of connection between actions and consequences, was measured at age eight using responses to 12 questions from the shortened version of the Nowicki-Strickland Internal-External (NSIE) scale. At age 16 it was measured using the 12 item Nowicki-Strickland Locus of Control Scale.

### Empathy

Empathy was measured at age seven using mother reported responses to five questions about the child’s attitudes towards sharing and caring.

### Impulsivity

Impulsivity was measured during two sessions at the age 8 direct assessment using a behaviour checklist. Testers rated whether the children demonstrated restlessness, impulsivity, fleeting attention, and lacking persistence. At age 11, the children were asked a battery of 10 questions designed to capture impulsive behaviour.

### Personality

Personality was measured at age 13 using the five-factor model of personality to capture five broad and independent dimensions of personality; the “Big Five” (extraversion, neuroticism, agreeableness, conscientiousness, and intellect) [38]. These were measured using self-report responses to 50 items of the International Personality Item Pool.

### Cognitive skills and outcomes

#### IQ

Intelligence was measured during the direct assessments at ages eight and 15 using the short form Wechsler Intelligence Scale for Children (WISC) from verbal, performance, and digit span tests and the Wechsler Abbreviated Scale of Intelligence (WASI) from vocabulary and matrix reasoning tests respectively. These assessments were administered by members of the ALSPAC psychology team overseen by an expert in psychometric testing. The short form tests have high reliability and the ALSPAC measures utilise subtests with reliability ranging from 0.70 to 0.96.

#### Educational achievement

We used four measures of educational achievement. The first three were average fine-graded point scores from three end of ‘Key Stage’ assessments during compulsory UK education at ages 11, 14 and 16. The fourth measure was a ranking of grades attained in post-compulsory A-levels at age 18, which are required for progression to university education. We used a measure of the three highest A-level grades grouped into ordered categories that is designed to provide a more fine-grained measure of A-level achievement than a pass/fail indicator [39]. This measure grades A-level results with the scores of A = 5, …, F = 0 and then combines the top three grades for each student (most UK children sit only 3 A-levels). At the time the cohort were studying, A-levels were non-compulsory and therefore all participants who did not continue into further education were set to missing. All measures were obtained through data linkage to the UK National Pupil Database (NPD) which represents the most accurate record of educational achievement available in the UK. All education data were extracted from the NPD Key Stage 4 (age 16) and Key Stage 5 databases (for further information see https://www.gov.uk/government/collections/national-pupil-database).

#### Employment

At age 23 participants were asked to report whether they were in full time paid employment of more than 30 hours per week, with responses coded as binary.

#### In education employment or training (EET)

Some participants may not be employed because they are still in full-time education or training, potentially skewing results by participation of the cohort in education or training. We therefore also used a binary measure to indicate whether a participant was in education employment or training at age 23. This measure was reverse coded so that a value of one reflects that a participant is in education employment or training, to be consistent with other outcomes.

#### Income

At age 23 participants were asked to report their take-home pay each month if they reported being in paid employment, with responses banded into the following categories: £1-£499; £500-£999; £1000-£1499; £1500-£1999; £2000-£2499; £2500-£3000; £3000 + .

#### Non-response

We also include a binary measure of questionnaire non-response at ages 18 and 24 to allow us to investigate correlations between noncognitive skills and cohort participation.

### Statistical analysis

We estimated phenotypic correlations between each measurement-pair using the Pearson’s correlation coefficient (*r*). Given the large number of associations explored (30 noncognitive skills; 2 cognitive skills; 7 socioeconomic outcomes; 2 non-response measures), all analyses were adjusted for false discovery rate using the Benjamini-Hochberg procedure [40].

Heritability of each occasion-specific noncognitive skill was estimated using genomic-relatedness-based restricted maximum likelihood (GREML) in the software package GCTA (see ref. [41] for a detailed description of this method). GCTA uses measured SNP level variation across all SNPs to estimate the genetic similarity between each pair of unrelated individuals in the sample. Univariate analyses were specified as:

1 $$\begin{array}{*{20}{c}} {y = X\beta + g + {\it{\epsilon }}} \end{array}$$

where *y* is the inverse normally rank transformed sex and age of measurement standardised measure of phenotype, *X* is a series of covariates indicating the first 20 principal components of inferred population structure to control for systematic differences in allele frequencies due to ancestral differences between different subpopulations (population stratification), *g* is a normally distributed random effect with variance $\sigma _g^2$ denoting the contribution of SNPs, and *∈* is residual error with variance $\sigma _{\it{\epsilon }}^2$. Heritability is then defined as the proportion of phenotypic variance that can be statistically explained by common genetic variation while holding inferred population structure constant:

2 $$\begin{array}{*{20}{c}} {\frac{{\sigma _g^2}}{{\sigma _g^2 + \sigma _{\it{\epsilon }}^2}}} \end{array}$$

To estimate the extent to which noncognitive traits share underlying genetic architecture we estimate genetic correlations between each phenotype-pair. Genetic correlations provide an estimate of the proportion of variance that two phenotypes share due to genetic variation (the overlap of genetic associations between two phenotypes). Genetic correlations were estimated as:

3 $$\begin{array}{*{20}{c}} {r_g = \frac{{\sigma _g^2\left( {A,B} \right)}}{{\sqrt {\sigma _g^2\left( A \right)\sigma _g^2\left( B \right)} }}} \end{array}$$

Where *r*_*g*_ is the genetic correlation between phenotypes *A* and *B*, $\sigma _g^2(A)$ is the genetic variance of phenotype *A* and $\sigma _g^2\left( {A,B} \right)$ is the genetic covariance between phenotypes *A* and *B*. To investigate associations between noncognitive and socioeconomic outcomes, we ran a series of multivariable regressions of age 16 educational achievement on each noncognitive skill, with all variables standardised to mean zero and standard deviation one, while controlling for sex, month of birth and cognition at age 8. Where repeated measurements of the same noncognitive skills exist (SDQ, communication, self-esteem, persistence, locus of control, impulsivity), we also estimate heritability and between-trait correlations using the mean value of the skill from all measurements. If traits are stable over time and measurement error is randomly distributed around the cross-sectional measures, mean values are expected to regress to the stable trait mean. All genetic analyses included the 20 principal components of population structure.

## Results

### How consistent are noncognitive skills over time?

**Fig. 1:**
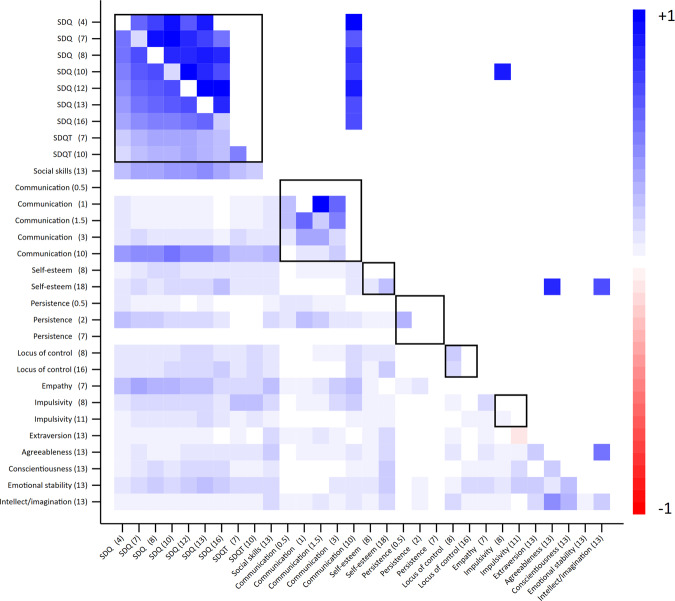
Heritability, phenotypic correlations and genetic correlations of noncognitive skills. Age (years) in parentheses. Values on the diagonal represent heritability; values below the diagonals represent phenotypic correlations; values above the diagonal represent genetic correlations. Multiple testing was handled using a False Discovery Rate threshold of 5%. Empty cells display correlations that fell below the False Discovery Rate threshold. Black outline boxes indicate the same skills measured at different occasions. SDQ: parent reported strengths and difficulties questionnaire; SDQT: teacher reported strengths and difficulties questionnaire. See Supplementary Tables 2 and 3 for full estimates.

Behavioural skills measured on the SDQ scale were consistent over time (parent reported SDQ *r* = 0.36 to 0.73, SE’s: < 0.02, mean *r* = 0.59; teacher reported SDQ *r* = 0.53, SE: 0.01), though consistency decreased with greater elapsed time between measures (Fig. 1, Supplementary Tables 2 and 3). Correlations between parent reported and teacher reported SDQ measures were lower (<0.4, SE’s: < 0.02, mean *r* = 0.31) than correlations within each measure. Correlations for other noncognitive measures over time were generally low (communication mean *r* = 0.13; self-esteem mean *r* = 0.14; persistence mean *r* = 0.11; locus of control mean *r* = 0.20; impulsivity mean *r* = 0.08) (Fig. 1), suggesting within-trait inconsistency (highly variable skills) or lack of reliability (high measurement error). Correlations amongst the Big Five personality types were low (mean *r* = 0.21, SE’s: < 0.02) except between the intellect/imagination and agreeableness subscales (*r* = 0.46, SE: 0.01). Phenotypic correlations for repeated measures of many noncognitive skills were generally positive (90% positive). Temporal phenotypic correlations were higher for cognitive measures of IQ (*r* = 0.60, SE: 0.01) and measures of educational achievement (*r* > 0.78 for compulsory education, SE’s: < 0.02) (Fig. 1, Supplementary Tables 2 and 3).

### How well do different noncognitive skills correlate?

**Fig. 2:**
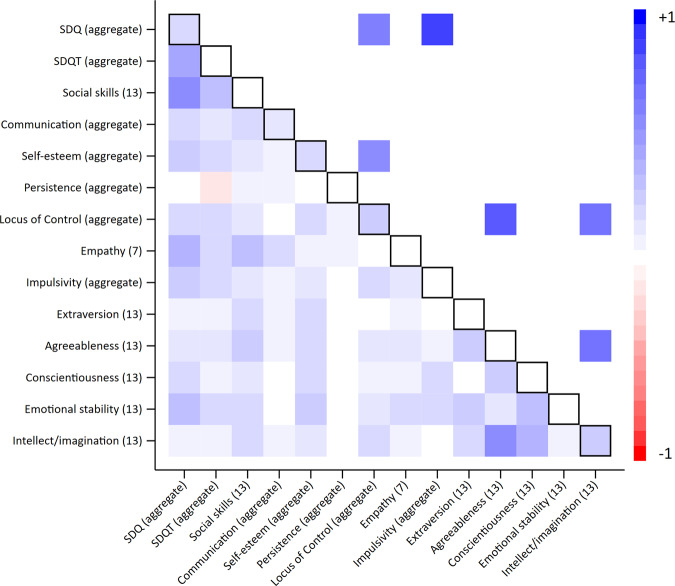
Heritability, phenotypic correlations and genetic correlations of aggregate noncognitive skills. Age (years) in parentheses; “mean” indicates aggregate measure of noncognitive skills measured more than once. Values on the diagonal represent univariate heritabilities; values below the diagonals represent phenotypic correlations; values above the diagonal represent genetic correlations. Multiple testing was handled using a False Discovery Rate threshold of 5%. Empty cells display correlations that fell below the False Discovery Rate threshold. Black outline boxes indicate the same skills measured at different occasions. SDQ: parent reported strengths and difficulties questionnaire; SDQT: teacher reported strengths and difficulties questionnaire. See Supplementary Tables 4 and 5 for full estimates.

Phenotypic correlations between measures of different noncognitive skills were generally low (mean between-skill *r* = 0.11) (Fig. 1 & Supplementary Figure 2). Parent-reported and teacher-reported SDQ was the only measure that correlated consistently with other noncognitive skills at *r* > 0.2 (SE’s: < 0.02). This between-trait correlation was strongest for social skills (*r* = 0.24 to 0.49, SE’s: < 0.02), communication at age 10 (*r* = 0.29 to 0.56, SE’s: < 0.02) and empathy (*r* = 0.17 to 0.38, SE’s: < 0.02). Between-skill phenotypic correlations were almost exclusively positive, suggesting that where patterns were observed, children who scored high on one noncognitive skill generally also scored highly on another. Correlations between the aggregate values of noncognitive skills that had been measured more than once were higher than for individual components but in general remained low (mean *r* = 0.11) (Fig. 2 and Supplementary Fig. 3). There was a weak negative correlation between the aggregate measures of persistence and teacher reported SDQ (*r* = −0.24; SE: 0.01). These correlations were driven by the persistence measure at age 6 months.

### Do noncognitive skills associate with socioeconomic outcomes?

**Fig. 3:**
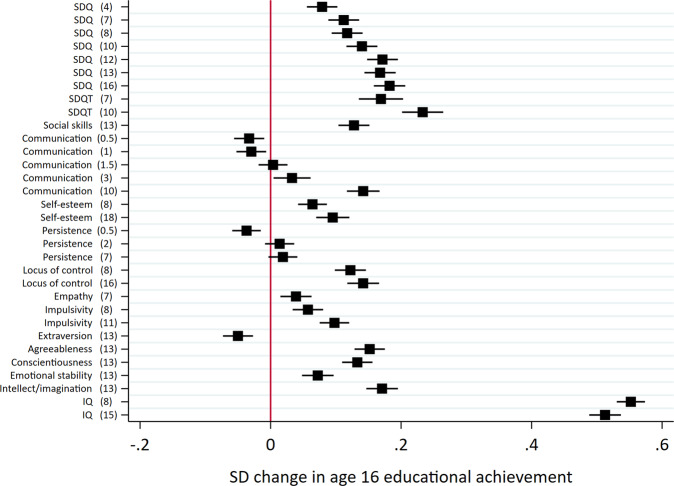
Associations between skills and educational achievement at age 16. All skills and educational achievement were standardised to have mean zero and standard deviation one. Models for noncognitive skills include sex, month of birth and IQ at age 8 as controls. Models for IQ include sex and month of birth as controls. Educational achievement measured as exam point score at age 16. Age (years) in parentheses. SDQ: parent reported strengths and difficulties questionnaire; SDQT: teacher reported strengths and difficulties questionnaire.

**Fig. 4:**
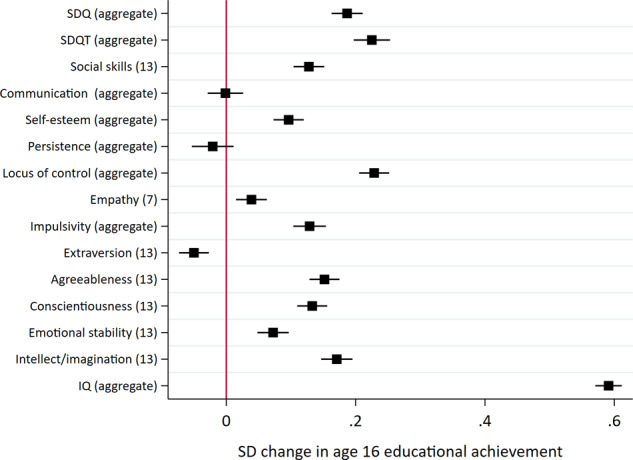
Associations between aggregate skills and educational achievement at age 16. All skills and educational achievement were standardised to have mean zero and standard deviation one. Models for noncognitive skills include sex, month of birth and IQ at age 8 as controls. Models for IQ include sex and month of birth as controls. Educational achievement measured as exam point score at age 16. Age (years) in parentheses. SDQ: parent reported strengths and difficulties questionnaire; SDQT: teacher reported strengths and difficulties questionnaire.

Results from the regression analyses are presented in Fig. 3. A one standard deviation (SD) increase in noncognitive skill measures was associated with a 0.05 SD decrease to 0.23 SD increase in educational achievement at age 16. There was considerable heterogeneity for estimates between skills and measurement occasions. In general, associations were larger for noncognitive skills measured later in childhood—closer in time to the educational measure—than those measured earlier in childhood. Associations were largest for teacher reported SDQ at age 10 (0.23, SE: 0.02). Modest associations with educational achievement were observed for social skills at age 13 (0.13, SE: 0.01), communication at age 10 (0.14, SE: 0.01), locus of control (age 8: 0.12, SE: 0.01); age 16: 0.14, SE: 0.01) and the agreeableness (0.15, SE: 0.01), conscientiousness (0.13, SE: 0.01) and intellect/imagination (0.17, SE: 0.01) scales of the Big Five. Associations between aggregate measures of the skills and socioeconomic outcomes (Fig. 4) were broadly consistent with the individual measures, with parent reported SDQ (0.18, SE: 0.01), teacher reported SDQ (0.23, SE: 0.01) and locus of control (0.23, SE: 0.01) having the largest associations with educational achievement. Associations between cognitive skills and educational achievement were consistently higher (age 8: 0.55, SE: 0.01; age 15: 0.51, SE: 0.01). These patterns of associations were consistent with educational achievement at other ages (Supplementary Fig. 4).

There was consistently strong evidence of associations between SDQ measures and labour market outcomes (Supplementary Figs. 5 and 6). For example, a one SD higher SDQ score at age 16 was associated with a 27% higher odds of being employed (OR: 1.27, 95% CI: 1.14, 1.42), a 53% higher odds of being in employment, education, or training (OR: 1.53, 95% CI: 1.30, 1.80), and a 0.12 SD (SE: 0.28) higher income. Associations for other noncognitive measures were less consistent, but there was strong evidence of association for all skills except persistence and the agreeableness and intellect/imagination scales of the Big Five. Noncognitive skills were inconsistently associated with questionnaire non-response later in life (Supplementary Fig. 7). Turning to aggregate measures of skills, associations were consistent with educational achievement across ages (Supplementary Fig. 8). There was strong evidence for associations with labour market outcomes for the parent and teacher reported SDQ, self-esteem and impulsivity (Supplementary Figs. 9 and 10). There was strong evidence that many aggregate measures were associated with lower odds of non-response (Supplementary Fig. 11). Aggregate communication and extraversion were both associated with higher odds of non-response.

### Are noncognitive skills heritable?

**Fig. 5:**
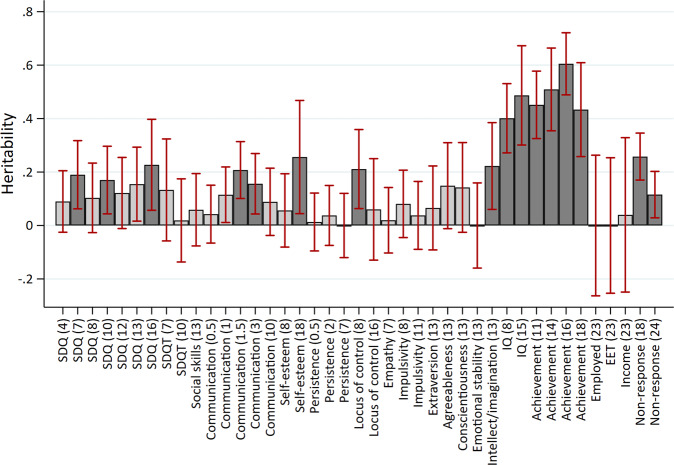
Heritability of skills and outcomes. Age (years) in parentheses. Dark shaded bars represent estimates below FDR threshold. Heritability estimated using GCTA-GREML on the full sample available for each phenotype. SDQ: parent reported strengths and difficulties questionnaire; SDQT: teacher reported strengths and difficulties questionnaire. EET: in education, employed or training. See Supplementary Table 8 for full estimates.

**Fig. 6:**
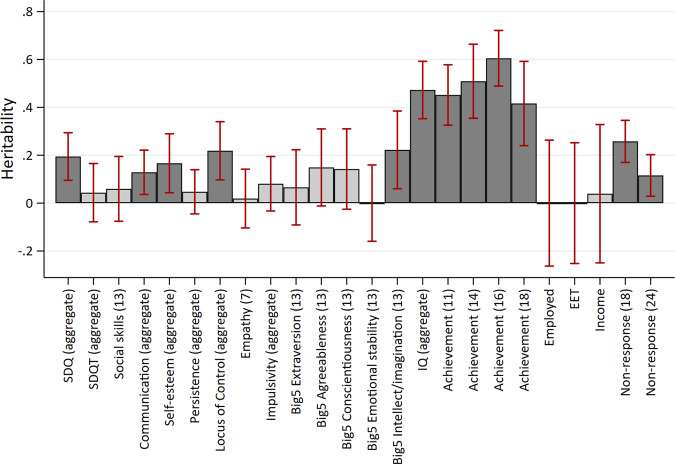
Heritability of aggregate skills and outcomes. Dark shaded bars represent estimates below FDR threshold. Heritability estimated using GCTA-GREML on the full sample available for each phenotype. SDQ: parent reported strengths and difficulties questionnaire; SDQT: teacher reported strengths and difficulties questionnaire. EET: in education, employed or training. See Supplementary Table 8 for full estimates.

Figure 5 displays the heritability estimates for all measures. There was strong evidence of non-zero heritability for the parent reported SDQ scale at multiple occasions (age 7 $h_{SNP}^2$: 0.19, SE: 0.07; age 10 $h_{SNP}^2$: 0.17, SE: 0.07; age 16 $h_{SNP}^2$: 0.23, SE: 0.08). There was little evidence for differences between the internalising and externalising subscales of the SDQ, though these estimates were imprecise (Supplementary Figure 12). Strong evidence of heritability was found for communication at both 18 months ($h_{SNP}^2$: 0.21, SE: 0.05) and 3 years ($h_{SNP}^2$: 0.16, SE: 0.06), self-esteem at 18 years ($h_{SNP}^2$: 0.26, SE: 0.11), locus of control at 8 years ($h_{SNP}^2$: 0.21, SE: 0.08), and the intellect/imagination subscale of personality type measured at 13 years ($h_{SNP}^2$: 0.22, SE: 0.08). Heritability of the mean value noncognitive skills was estimated with greater precision (Fig. 6), with strong evidence for non-zero heritability for the mean responses of SDQ, ($h_{SNP}^2$: 0.20, SE: 0.05), communication ($h_{SNP}^2$: 0.13, SE: 0.05), self-esteem ($h_{SNP}^2$: 0.17, SE: 0.06), and locus of control ($h_{SNP}^2$: 0.22, SE: 0.06) (Fig. 5). The heritability of cognitive skills was higher at 0.4 (SE: 0.07) at age 8 and 0.49 (SE: 0.10) at age 15. Educational outcomes were highly heritable ($h_{SNP}^2$ > 0.4) but there was little evidence of heritability for the labour market outcomes. Heritability of questionnaire non-response was estimated at 0.26 (SE: 0.05) and 0.12 (SE: 0.04) at ages 18 and 24 respectively.

### Do noncognitive skills have a shared genetic architecture?

There was strong evidence for within trait genetic correlations over time for the parent reported SDQ measures (*rG*: 0.62 to 1.00, mean *rG:* 0.81), and communication at age one, 18 months and age 3 (mean *rG*: 0.72). There was very limited evidence for genetic correlations across different noncognitive skills (Fig. 1, above the diagonal), though estimation precision was low for most skills given the low SNP heritabilities. Genetic correlations between different noncognitive measures were only observed between the parent reported SDQ measures and communication at age 10 (*rG*: 0.69 to 0.91, mean *rG: 0.80*). This compares to near-unity genetic overlap for IQ at ages 8 and 15 (*rG*: 0.97, SE: 0.08). Using mean values of noncognitive skills that were measured at multiple occasions (Fig. 2) there was strong evidence for non-zero genetic correlations between SDQ and locus of control (*rG*: 0.50, SE: 0.17), SDQ and impulsivity (*rG:* 0.76, SE: 0.22), and self-esteem and locus of control (*rG*: 0.50, SE: 0.2).

There was strong evidence for genetic correlations between educational achievement and the SDQ measures (*rG*: 0.44 to 0.71, mean *rG:* 0.54), communication at age 10 (*rG*: 0.76 to 1, mean *rG:* 0.92), self-esteem at age 18 (*rG*: 0.07 to 0.50, mean *rG:* 0.4), the agreeableness (*rG*: 0.65 to 0.81, mean *rG:* 0.72) and the intellect/imagination (*rG*: 0.67 to 0.73, mean *rG:* 0.72) subscales of the Big Five personality types. There was strong evidence for non-zero genetic correlations with IQ and education for the mean values of parent SDQ, teacher SDQ and locus of control. There was little evidence of genetic correlations between labour market outcomes and any of the noncognitive or cognitive skills.

## Discussion

Our results provide longitudinal evidence into the consistency of a large range of noncognitive skill measures throughout childhood and adolescence. Measures of behavioural and communication skills as captured by the SDQ correlated strongly phenotypically (mean *r* = 0.59) and genotypically (mean *r* = 0.81) over time. Correlations of other measured noncognitive skills over time were low, contrasting with previous research that has evidenced temporal stability of noncognitive skills [21, 22]. This heterogeneity in consistency may be due to the accuracy with which the measures used were able to reliably measure underlying noncognitive skills. For example, the SDQ scale used responses to a large battery of validated questions which may be more reliable than measures of impulsivity assessed from child behaviour during direct assessments. Furthermore, results using aggregated values of noncognitive skills were more precise than those using individual measures. By incorporating information from multiple occasions, aggregate measures are expected to contain less measurement error than individual measures. However, combining longitudinal measures also presents limitations as developmental differences in measures by age, which may be important in childhood, will be averaged out. Where data from multiple raters or sources is present, combing these may also provide measures of noncognitive skills that contain less error [42]. Future research proposing more detailed measurement of noncognitive skills may therefore improve the ability of measures to capture underlying skills. Similarly, analytical approaches that are well equipped to model composite or latent traits, such as structural equation modelling, may be useful for testing the longitudinal consistency of noncognitive skills [17, 18]. The lack of consistency in noncognitive skills over time may have also reflected genuine intra-individual temporal variation in these skills over time (e.g. due to schooling) or that these noncognitive skills are themselves not robust constructs. It is important to note there is some variation in the measurement of skills that we used. This is somewhat unavoidable as skill measurements vary depending on the age at which a child is assessed, but measurement artefact may nevertheless reflect some of the variation in measurement. Noncognitive skills have been argued as promising targets for interventions to improve outcomes because they are potentially more modifiable than cognitive skills [1, 11, 22]. However, our results question how stable some of these skills are and how accurately they may be measured.

Correlations between different noncognitive skills were weak, supporting the notion that these constructs capture empirically different psychosocial phenomena rather than a single underlying or latent noncognitive factor. The SDQ scale was the only measure to consistently correlate with other noncognitive skills, agreeing with a previous study that found low phenotypic correlations between different noncognitive skills [9]. Stronger between-trait correlations were observed using aggregate values of the noncognitive skills, which again may reflect greater measurement precision. The cognitive measures we used were more strongly correlated over the same period, though this may reflect that they were assessed using widely validated measures. Future studies that combine longitudinal data on noncognitive skills with multi-source multi-method approaches may be able to better elucidate relationships between noncognitive skills.

We found limited evidence of genetic correlations within or between noncognitive skills, with only the parent reported SDQ demonstrating consistent genetic architecture over time. This may reflect the influence of shared parent-offspring genetics on reporting, or parental genetics influencing offspring noncognitive skills indirectly through dynastic effects [43] (there would be no such shared teacher-student genetics). Twin studies have found strong genetic correlations between both observed and latent noncognitive and cognitive skills [17, 18, 28, 29], but our results did not support this. The differences between these results and previous studies may have arisen due to differences in the quality of noncognitive skill measurements; differences due to cross-sectional and longitudinal study designs; differences in analytical approach [17, 18]; reporting bias by the study mothers (for example where their child performed poorly); differences in the study populations; younger participants than previous studies combined with lower heritability of measures in adolescence than in adulthood [44]; or selective reporting and the use of samples or measures of convenience in previous studies [20]. While we reported a range of noncognitive skills in ALSPAC, many previous studies have reported only one or a small number of noncognitive skills.

Our results also showed that associations between noncognitive skills and socioeconomic outcomes were generally weak, contradicting findings from previous individual studies [1, 4, 11] but supporting a recent systematic review [20]. Behavioural problems as captured by the SDQ scale, social skills, and locus of control were the only noncognitive measures to phenotypically associate with educational outcomes strongly and consistently. Associations were stronger between educational achievement and the teacher than the parent reported measures of the SDQ scale. This may suggest that teachers more objectively identify education related problematic behaviour than parents, or that teachers’ response to their pupils directly influences their educational outcomes. Noncognitive skills capture a diverse range of characteristics and may have varying relevance to educational and labour market outcomes; further longitudinal research is required to better elucidate these relationships. Neither noncognitive nor cognitive skills associated strongly with the labour market outcomes measured here. While this contrasts with previous studies [1, 11], our labour market outcomes were observed soon after entry to the labour market and therefore may have more closely resembled institutional effects than noncognitive skills. Some have argued that noncognitive skills and personality traits are as important as cognitive skills for many dimensions of behaviour and socioeconomic outcomes [7], but our results do not support this. The standardised effect sizes of noncognitive skills were at most a third that of cognitive skills.

Many noncognitive and cognitive skills were weakly negatively correlated with non-response phenotypically, suggesting that individuals who scored low on these skills were more likely to later drop out of the ALSPAC study. This may have important implications for participant representativeness and generalisability in cohort studies. Cognitive ability and achievement were negatively genotypically correlated with non-response, adding to the growing body of evidence that study non-response is genetically patterned [45].

The SDQ scale was the only noncognitive measure for which we consistently found strong evidence of heritability (~0.15), consistent with results from previous studies looking at behavioural problems [46, 47]. Non-zero heritability has been estimated for the other non-cognitive skills used here, though this has been based on twin studies which are able to exploit all genetic variation [48–50]. Heritability and stability depend on the developmental period being examined and so comparison of estimates is difficult. Previous research has demonstrated that the heritability of cognitive ability rises with age while the heritability of personality traits decreases with age [51], but our data did not reflect this. Many of the genetic correlations we observed were imprecise due to the low heritability estimates, but our estimates suggested an upper bound heritability of 0.3 for most noncognitive skills.

This study has several limitations. First, it is possible that measurement error was unusually high in the noncognitive measures used in the ALSPAC study. However, the measures used in ALSPAC have been widely validated are consistent with those used in previous studies [10]. Furthermore, measurement error would need to have been high across all measures used from birth to age 18 so to explain these results. Our findings were consistent when using aggregated measures of noncognitive skills where they had been measured at least twice, suggesting that our results were not driven by differential measurement error across occasions. Future studies into test-retest reliability of noncognitive skills based on different longitudinal samples could help resolve these questions. Second, many of the genetic correlations were estimated with extremely low precision, often being constrained at the values of −1 or 1 (see supplementary Table 2). This is likely due to the low estimated heritability of the noncognitive skills. Low heritability implies a small genotypic contribution (either in the number of individual variants associated with a trait or the strength of these associations) and therefore lower power to detect genetic correlations between skills. Future studies conducted on larger samples are required to more accurately the estimate heritability of, and genetic correlations between noncognitive skills and other phenotypes. Third, our genetic associations may have been biased by uneven linkage disequilibrium, residual population structure, or assortative mating [43, 52, 53]. We controlled for the first twenty principle components of population structure to account for population structure but this may not have accounted for all differences [54]. Assortative mating is thought to be low for noncognitive traits [25], but will have inflated our genetic associations if present [55]. It is possible that assortment on noncognitive skills may be negative and future work is required to determine this. Fourth, the use of labour market outcomes at age 23 may mean that some of our study participants have not yet transitioned into their stable career employment. While our use of an education, employment and training outcome variable reduces the problem that those still in education or unemployed have not yet entered the labour market, it does not provide any indication that employed study participants have entered desired or long-term employment. Finally, it is possible that some of the measures could reflect parental rather than child genes where they are parent rather than self-reported.

In conclusion, our results highlight that noncognitive skills are likely to be highly heterogenous. Measures of noncognitive skills were varied both over time and across different measures in the same individuals, but some individual measures such as the SDQ demonstrated strong internal consistency throughout childhood. Furthermore, many noncognitive measures associated weakly with educational and employment outcomes at entry to the labour market, particularly when measured early in childhood.

## Supplementary information

Supplementary Material

Supplementary Tables

## Data Availability

The code used to analyse these data are available at https://github.com/timtmorris/ALSPAC_noncognitive_consistency.
